# High‐Efficiency Fullerene Solar Cells Enabled by a Spontaneously Formed Mesostructured CuSCN‐Nanowire Heterointerface

**DOI:** 10.1002/advs.201700980

**Published:** 2018-02-02

**Authors:** Wai‐Yu Sit, Flurin D. Eisner, Yen‐Hung Lin, Yuliar Firdaus, Akmaral Seitkhan, Ahmed H. Balawi, Frédéric Laquai, Claire H. Burgess, Martyn A. McLachlan, George Volonakis, Feliciano Giustino, Thomas D. Anthopoulos

**Affiliations:** ^1^ Department of Physics Imperial College London South Kensington London SW7 2AZ UK; ^2^ Division of Physical Sciences and Engineering, KAUST Solar Centre King Abdullah University of Science and Technology (KAUST) Thuwal 23955‐6900 Saudi Arabia; ^3^ Department of Materials Faculty of Engineering Imperial College London South Kensington London SW7 2AZ UK; ^4^ Department of Materials University of Oxford Parks Road Oxford OX1 3PH UK; ^5^ Department of Materials Science and Engineering Cornell University Ithaca NY 14850 USA

**Keywords:** copper (I) thiocyanate, fullerenes, mesostructured heterointerfaces, PCBM, solar cells

## Abstract

Fullerenes and their derivatives are widely used as electron acceptors in bulk‐heterojunction organic solar cells as they combine high electron mobility with good solubility and miscibility with relevant semiconducting polymers. However, studies on the use of fullerenes as the sole photogeneration and charge‐carrier material are scarce. Here, a new type of solution‐processed small‐molecule solar cell based on the two most commonly used methanofullerenes, namely [6,6]‐phenyl‐C61‐butyric acid methyl ester (PC_60_BM) and [6,6]‐phenyl‐C71‐butyric acid methyl ester (PC_70_BM), as the light absorbing materials, is reported. First, it is shown that both fullerene derivatives exhibit excellent ambipolar charge transport with balanced hole and electron mobilities. When the two derivatives are spin‐coated over the wide bandgap p‐type semiconductor copper (I) thiocyanate (CuSCN), cells with power conversion efficiency (PCE) of ≈1%, are obtained. Blending the CuSCN with PC_70_BM is shown to increase the performance further yielding cells with an open‐circuit voltage of ≈0.93 V and a PCE of 5.4%. Microstructural analysis reveals that the key to this success is the spontaneous formation of a unique mesostructured p–n‐like heterointerface between CuSCN and PC_70_BM. The findings pave the way to an exciting new class of single photoactive material based solar cells.

## 1 Introduction

The performance of the organic bulk heterojunction (BHJ) solar cells has been increasing steadily over the last few years, and has reached 13% for the single‐junction solar cells.^1^, ^2^, ^3^ However, these high efficiencies have come at the cost of an increase in the complexity of the cells, where often finely tuned nanomorphologies are required, which renders them both less stable and reproducible, along with difficult‐to‐synthesize polymers and small molecules with high associated production costs.^4^, ^5^, ^6^ In addition, the inherent trade‐off between the short‐circuit current (*J*_sc_) and the open‐circuit voltage (*V*_OC_), due the requirement for the lowest unoccupied molecular orbital offset between the donor and the acceptor to be more than 0.3 eV, is thought to be a limiting factor in pushing cell efficiencies to above 15%.^7^ Owing to the inherent disadvantages associated with the BHJ cell architecture, there has been a recent drive to move toward cell architectures which make use of only a single active layer, with the active material fulfilling the simultaneous roles of light absorption, exciton dissociation, and charge transport, in a manner similar to the emerging hybrid metal halide perovskite cells. A move toward such an architecture should reduce the complexity of the cell fabrication, increasing stability and potentially lowering the production cost.

Among the organic materials previously incorporated into single cell architectures are diblock copolymers and donor‐acceptor cooligomers.^8^, ^9^, ^10^, ^11^ However, intrinsic difficulties with excessive recombination and charge transport due to the extreme proximity of donor and acceptor molecules have limited the power conversion efficiencies (PCEs) of such attempts to below 3%. In contrast, more significant advancements have been made using the so‐called Schottky junction fullerene solar cells, in which small concentrations, usually around 5%, of donor materials are incorporated into the fullerene matrix, which acts as the main absorber. Such cell architectures have shown to exhibit high *V*_OC_ of over 1 V, and efficiencies of up to 6%.^12^, ^13^, ^14^, ^15^, ^16^, ^17^, ^18^, ^19^, ^20^, ^21^ The importance of these cells cannot be understated; fullerenes have been the prototypical 3D semiconductor with a molecular symmetry that is unmatched by any other molecular semiconducting material, and have been synonymous with the rise of the third generation photovoltaics in the form of electron acceptors. Furthermore, the apparent ability of fullerenes to suppress hysteresis in the recently emergent metal halide perovskite solar cells,^22^, ^23^, ^24^, ^25^, ^26^, ^27^ leading to high open‐circuit voltages^28^, ^29^, ^30^ and fill factors,^31^ have made fullerenes even more attractive and technologically relevant.

Here, we show that the high‐efficiency solar cells can indeed be realized using two of the most commonly used fullerene derivatives, namely [6,6]‐phenyl‐C61‐butyric acid methyl ester (PC_60_BM), and [6,6]‐phenyl‐C71‐butyric acid methyl ester (PC_70_BM), as the light absorbing materials. Key to our success is the incorporation of the wide bandgap p‐type semiconductor copper thiocyanate (CuSCN), as the hole‐transporting, electron‐blocking material.^32^, ^33^ In addition to its excellent and strictly unipolar transport properties, CuSCN is a superb hole‐transport layer (HTL) choice due to its low cost, toxicity, and annealing temperature, its chemical stability and its compatibility with a range of solution‐processing methods.^32^ While poor solubility in solvents other than sulfur‐based ones has thus far limited its use in environments other than strictly controlled laboratory conditions, a recent report on the fabrication of a variety of optoelectronic devices based on CuSCN dissolved in aqueous ammonia may open the door to its large scale application.^34^ Here, among the two methanofullerenes used in the solar cells in conjunction with CuSCN, PC_70_BM is found to exhibit the highest performance due to its enhanced absorption coefficient. Specifically, we show that the bilayer CuSCN/PC_70_BM solar cells yield high *V*_OC_ of up to 0.95 V leading to maximum PCE values of 1%. Mixing the CuSCN with PC_70_BM is found to enhance the cell's PCE further reaching a maximum value of ≈5.4%. Electrical and microstructural characterization of the materials and devices combined with density functional theory (DFT) calculations, suggest that the main factors responsible for the excellent performance are: (i) efficient charge extraction enabled by the highly balanced hole and electron transport within the fullerene component, (ii) the ability of splitting tightly bound Frenkel excitons at the CuSCN:fullerene interface due to band offset, and (iii) the existence of a unique spontaneously formed mesostructured CuSCN‐nanowire:fullerene heterointerface.

## 2 Results and Discussion

**Figure 1:**
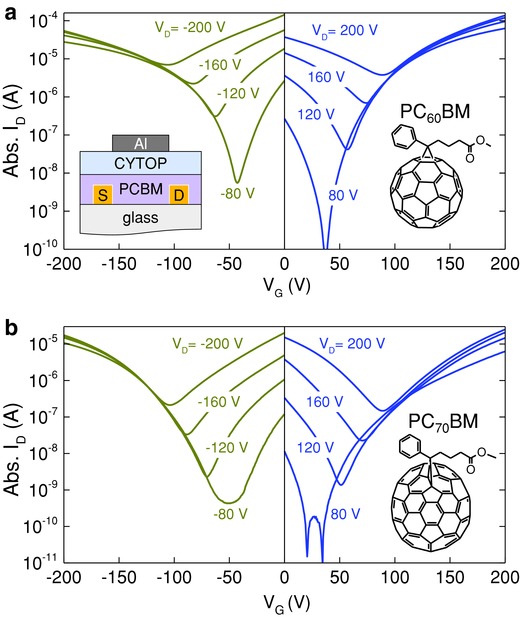
Transfer characteristics of a) PC_60_BM and b) PC_70_BM TG‐BC transistors employing CYTOP as the dielectric, at various source–drain voltages. The channel lengths (*L*) and widths (*W*) of the transistors are 30 µm and 1 mm, respectively. The device architecture is shown in the top left inset, with the chemical structures of PC_60_BM and PC_70_BM also shown in the insets on the right.

An essential property of the photoactive layer of a solar cell is for it to be able to transport both holes and electrons. In order to study the charge transport properties of PC_60_BM and PC_70_BM, top‐gate, bottom‐contact (TG‐BC) thin‐film transistors (TFTs) were fabricated (**Figure** **1**a, inset) using previously reported procedures (see the Experimental Section). Figure 1 displays representative transfer characteristics measured for a PC_60_BM (1a) and a PC_70_BM (1b) transistor with channel length (*L*) and width (*W*) of 30 and 1000 µm, respectively. Both transistors exhibit balanced ambipolar characteristics as evident by the comparable current levels measured in the p‐channel and n‐channel operating regimes. **Table** **1** summarizes the hole (*µ*_h(s)_) and electron (*µ*_e(s)_) mobility values measured in saturation for both transistors as well as values reported in the literature.^35^, ^36^, ^37^, ^38^ For PC_70_BM, the calculated *µ*_h(s)_ is the highest reported to date and surpasses the only value found in the literature (2 × 10^−5^ cm^2^ V^−1^ s^−1^)^39^ by more than three orders of magnitude. Similarly, the *µ*_h(s)_ value extracted for PC_60_BM is also the highest reported to date and exceeds that reported by Anthopoulos and co‐workers^35^ by more than one order of magnitude. The slightly higher electron mobility measured for both methanofullerenes is not believed to be an intrinsic property of the two molecules but is most likely attributed to the existent of larger injection barrier for holes as compared to electrons.

**Table 1:** Summary of the charge mobility values for PC_60_BM and PC_70_BM reported in the literature and measured in this work

Semiconductor	Carrier type	Mobility [cm^2^ V^−1^ s^−1^]	Method	TFT architecture	Dielectric	Ref.
PC_60_BM	*e*	2 × 10^−1^	FET	BG‐TC	BCB	34
	*e*	4.2 × 10^−1^	FET	TG‐BC	CYTOP	This work
	*h*	8 × 10^−3^	FET	BG‐BC	SiO_2_/HMDS	35
	*h*	1.8 × 10^−1^	FET	TG‐BC	CYTOP	This work
PC_70_BM	*e*	1.2 × 10^−1^	FET	TG‐BC	CYTOP	This work
	*e*	1 × 10^−2^	FET	BG‐TC	SiO_2_/HMDS	36
	*e*	1 × 10^−3^	SCLC	N/A	N/A	37
	*h*	2 × 10^−5^	FET	BG‐BC	SU8	38
	*h*	9 × 10^−2^	FET	TG‐BC	CYTOP	This work

On the basis of these results, we can unambiguously conclude that PC_60_BM and PC_70_BM are excellent ambipolar semiconductors with well‐balanced hole and electron mobilities. The implications of these findings are significant for the general field of organic photovoltaics where the contribution to the cell's hole transport properties by the acceptor molecules has not yet been considered extensively, with only a handful of exceptions.^40^ Clearly, however, this contribution cannot anymore be neglected, since the hole mobility in both PC_60_BM and PC_70_BM appears to be significantly higher than previously assumed, and for most cases, if not all, surpass the mobility values of the donor polymers used in the best‐performing organic BHJ solar cell.^3^

**Figure 2:**
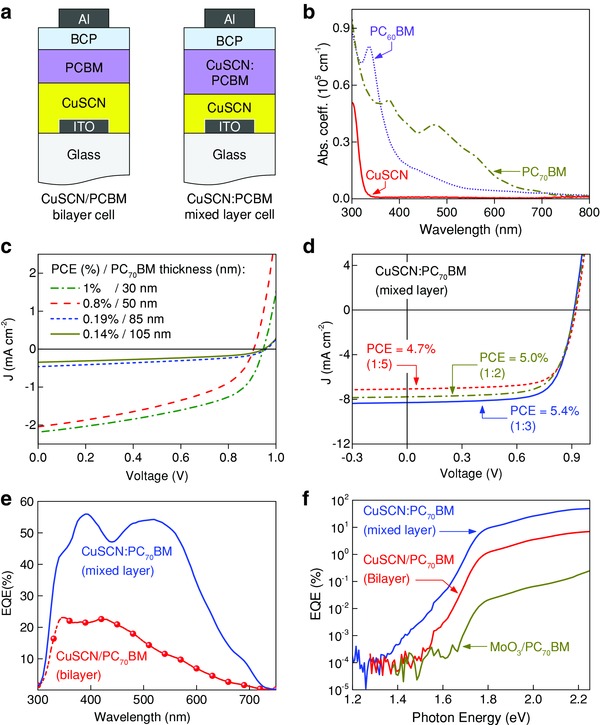
a) Schematic of device architecture of a bilayer (left) and a mixed layer CuSCN/PCBM (right) solar cell. b) The absorption spectra of CuSCN, PC_60_BM, and PC_70_BM. The current–voltage curves under illumination of c) the bilayer CuSCN/PC_70_BM solar cells with different PC_70_BM layer thicknesses and d) the mixed layer CuSCN:PC_70_BM solar cells with different CuSCN:PC_70_BM ratios (by weight). e) The external quantum efficiency (EQE) spectra between 300 and 700 nm of the best performing bilayer and mixed layer cells, and f) the sub‐bandgap EQE showing the absorption tail of the bilayer and mixed layer cells as well as of a MoO_3_/PC_70_BM bilayer cell for comparison.

To investigate whether the two methanofullerenes could be used as the sole photogeneration material, two different types of solar cells were fabricated based on a bilayer and a mixed layer configuration (**Figure** **2**a). In both cells, the role of CuSCN is to selectively transport holes to the anode while blocking electrons and hence minimizing recombination losses at the semiconductor/anode interface.^34^, ^41^, ^42^, ^43^, ^44^, ^45^, ^46^, ^47^ Bathocuproine (BCP) was chosen as the electron transport layer because of its superior ability to block holes while facilitating electron extraction by the cathode electrode.^48^, ^49^

Prior to device fabrication, the absorption characteristics of all materials used to fabricate the cells were studied. Figure 2b shows the thin film UV–vis optical absorption spectra of PC_60_BM, PC_70_BM, and CuSCN. In agreement with previously published results, the spectra show that PC_60_BM and PC_70_BM films are absorptive in the visible region, while the CuSCN film is absorptive in the ultraviolet region.^32^, ^42^ The PC_60_BM film shows significantly lower absorption coefficient between 400 and 600 nm compared to the PC_70_BM film, which exhibits a relatively high absorption coefficient between 350 and 600 nm (≈3.9 × 10^4^ cm^−1^). Therefore, emphasis was placed on investigating the use of PC_70_BM as the photoactive element in the solar cells of various configurations.

Figure 2c shows the current density–voltage (*J*–*V*) characteristics for the bilayer CuSCN/PC_70_BM solar cells of different PC_70_BM thicknesses. The measured PCE increases from 0.13% when the thickness of the PC_70_BM layer is 105 nm to 1% when thickness is reduced to an optimal value of 30 nm. The bilayer cell exhibits a high *V*_oc_ of up to 0.95 V, that is comparable to previously reported values for the fullerene‐only Schottky‐junction solar cells.^12^ For the optimized device (30 nm), the extracted values for FF = 47% and *J*_SC_ = 2.2 mA cm^−2^ are also comparable to previous results measured for the single semiconductor solar cells,^18^ suggesting that both reasonable charge photogeneration and transport within the methanofullerene layer occur. Indeed, a closer look at the external quantum efficiency (EQE) spectra of the bilayer cell (Figure 2e) reveals that the charge generation occurs across the entire absorption spectrum of PC_70_BM. The rather efficient photogeneration is most likely facilitated through the charge transfer across the critical CuSCN/PC_70_BM heterointerface (Figure S1, Supporting Information). It is noteworthy that here PC_70_BM acts as the hole donor species, rather than the electron acceptor, and excitons dissociation and charge transfer occur at a hybrid CuSCN/methanofullerene interface, most likely driven by the offset between the valence band of CuSCN and the highest occupied molecular orbital of PC_70_BM. This type of charge generation in donor/acceptor systems has previously been termed the “channel II” charge generation pathway, with “channel I” referring to the photoexcited electron transfer from the donor to the acceptor; until recently, the design of donor/acceptor systems has been focused on optimizing channel I charge generation, often overlooking the contribution from channel II contribution. Note that in the present case, we assume that charge generation proceeds almost entirely via channel II generation due to the large bandgap and high lying conduction band of CuSCN.^50^, ^51^ It is also noteworthy that this is in contrast to other studies of hybrid inorganic–organic donor–acceptor systems, which have almost exclusively studied p‐type polymeric donor materials with n‐type metal oxide acceptors.^52^, ^53^, ^54^, ^55^, ^56^, ^57^, ^58^

Having established that charge generation at the CuSCN/PC_70_BM interface occurs, we investigated the possibility of increasing the interface area between the p‐type (CuSCN) and the n‐type (PC_70_BM) layers, by physically blending the CuSCN and PCBM formulation prior to spin‐coating (see the Experimental Section). The *J*–*V* characteristics of the mixed‐phase CuSCN:PC_70_BM device for different ratios are shown in Figure 2d. In general, increasing the amount of CuSCN in the blend of PC_70_BM and CuSCN improves the charge photogeneration, with the optimized CuSCN:PC_70_BM ratio being 1:3 (CuSCN concentration 40 mg mL^−1^). While the cells exhibit a slightly reduced *V*_OC_, as compared to the bilayer device, both the *J*_SC_ and the FF are dramatically improved, reaching values of 8.3 mA cm^−2^ and 72%, respectively, resulting in an average PCE of 5.1 ± 0.2% and a maximum value of 5.4%. To this end we emphasize that devices with PCEs > 6% have been fabricated, but were especially difficult to reproduce. Despite this, the obtained results are remarkable if one considers the semitransparent nature of the best performing cells (see Figure S2, Supporting Information). A similar improvement was observed in CuSCN:PC_60_BM cells (Figure S3, Supporting Information), albeit with much lower efficiency due to the lower optical absorption of PC_60_BM and its lower solubility.

The quantum efficiency spectra of optimized CuSCN:PC_70_BM devices are shown in Figure 2e. The trend of *J*_SC_ value measured for these devices (Table S1, Supporting Information) is consistent with the EQE spectra (Figure 2e). In particular, an integrated photocurrent of 7.6 mA cm^−2^ for the mixed layer CuSCN:PC_70_BM cells is in agreement (±0.3 mA cm^−2^; ±5%) with the *J*_SC_ value provided in Table S1 (Supporting Information). This device also shows the contribution of PC_70_BM to the EQE is in the range 350–600 nm, and reaching a maximum EQE ≈ 55% at wavelengths of 400 and 510 nm. In order to investigate how the charge is generated in the CuSCN:PC_70_BM device, we performed sub‐bandgap EQE measurements. This technique can be used to study the contribution of sub‐bandgap photocurrent produced by the direct formation of the charge‐transfer (CT) states upon long wavelength illumination of the ground state CT absorption, and hence provides insight on whether the charges are generated within the PC_70_BM and/or at the CuSCN/PC_70_BM interface. The subgap‐EQE spectrum of the mixed layer CuSCN:PC_70_BM and the bilayer CuSCN/PC_70_BM devices are shown in Figure 2f. For comparison, a spectrum of a bilayer device where the CuSCN has been replaced with a different hole transporting material, namely molybdenum trioxide (MoO_3_), is also shown. Since CuSCN absorbs only at photon energies above ≈3.5 eV, it is assumed that all photocurrent generated at photon energies ≥1.7 eV is due to absorption in the PC_70_BM. Interestingly, both the bilayer and mixed phase CuSCN based devices show an extended absorption tail that does not overlap with the EQE of the MoO_3_/PC_70_BM device or with the absorption spectrum of CuSCN. We attribute this to the low‐energy absorption of CT‐like states created at the interface between CuSCN and PC_70_BM, resembling those formed in the conventional organic BHJ solar cells.^59^ Note that in the case of the bilayer device the absorption tail does not extend to as low energies as in the mixed layer device, which is likely due to the reduced number of CuSCN–PC_70_BM interfaces in the bilayer case compared to the mixed layer device resulting in a lower density of CT‐like states.

**Figure 3:**
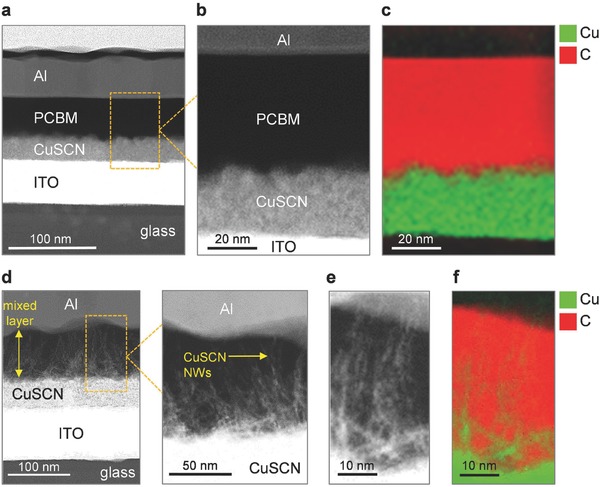
a) Cross‐sectional transmission electron microscopy (TEM) images of a bilayer CuSCN/PC_70_BM cell and b) a higher resolution scanning TEM (STEM) of the CuSCN/PCBM interface along with c) an elemental mapping of Cu and C of the same interface using electron loss spectroscopy (EELS). d) TEM image of a mixed layer CuSCN/PC_70_BM cell, with the CuSCN/PC_70_BM interface magnified to highlight the protruding CuSCN nanowires (NWs) into the PC_70_BM. e) A higher magnification image and f) elemental mapping reveals the length of the nanowires to be in the range of 30–100 nm.

In an effort to understand the origin of the difference in the performance characteristics between the bilayer CuSCN/PC_70_BM and the mixed layer CuSCN:PC_70_BM cells, we analyzed the microstructure of the active layers using high‐resolution transmission electron microscopy. **Figure** **3**a shows a cross‐sectional TEM image of the bilayer CuSCN/PC_70_BM cells. As expected every layer is clearly defined with little intermixing between the different material components. Figure 3b shows a higher resolution scanning TEM (STEM) image of the heterojunction, while Figure 3c displays the elemental map for Cu and C obtained via electron energy loss spectroscopy (EELS). The latter provides direct proof of the spatial distribution of Cu and C indicative of the presence of a well‐defined CuSCN/PC_70_BM bilayer. Surprisingly, in the mixed layer CuSCN:PC_70_BM cell (Figure 3d), the formation of the randomly oriented CuSCN‐nanowires (CuSCN NWs) can be seen. These NWs appear to protrude from the compact CuSCN layer beneath and extend into the PC_70_BM‐dominated region of the mixed layer above. The length of these spontaneously formed CuSCN NWs varies and is found to be in the range of 30–100 nm (Figure 3d,e). An elemental mapping performed via EELS on the same device provides further proof of the radically different distribution of Cu across the two cells (Figure 3f). Based on these findings, we conclude that the presence of the spontaneously formed CuSCN nanowires is the main reason for the dramatically enhanced *J*_SC_ and supports the idea that this unique mesostructured CuSCN/methanofullerene p–n heterointerface is responsible for the improved carrier photogeneration. Finally, the noticeable increase in the FF suggests that this mesostructured heterointerface underpins the observed improvements in the carrier transport/collection, a conclusion supported by the increased shunt (from 13 to 48 kΩ) and reduced series (from 430 to 90 Ω) resistance in the mixed CuSCN:PC_70_BM based cells.

**Figure 4:**
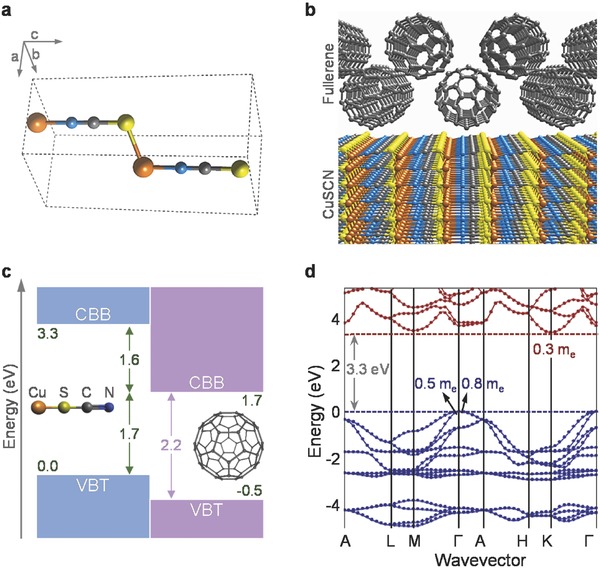
a) Unit cell of β‐CuSCN. b) Atomistic model of the interface between the (11$\bar{2}$0) surface β‐CuSCN and C_60_ absorbed over the C—N surface bond. c) The resulting band level alignment of the CuSCN/C_60_ interface calculated via DFT‐HSE. The energy scale is aligned to the CuSCN valence band top, all energies are in eV. d) DFT‐HSE electronic band structure of β‐CuSCN. The bandgap is calculated at 3.3 eV, and the effective masses for the electron holes along the molecular *c*‐axis are 0.3*m*_e_ and 0.8*m*_e_, respectively. The effective mass for the light holes along ΓM is 0.5*m*_e_. (Cu: orange spheres, S: yellow, C: gray, and N:blue.)

To further elucidate the atomic‐scale mechanisms at the interface between the fullerene and CuSCN, we employed calculations from first‐principles. We model the interface using the stable crystal β‐phase of CuSCN^60^ with the unit cell as shown in **Figure** **4**a and the pristine C_60_ and C_70_ crystals, because of the uncertain position of the functional group of the methanofullerenes. The functional group impacts the molecules solubility and is known to have practically no impact on their electronic properties, and hence our results do not depend on the presence and position of the group. Nonpolar (11$\bar{2}$0) of β‐CuSCN, which is the most energetically favorable surface, has been shown to have a very small formation energy.^60^ Three possible absorption sites on the CuSCN surface were considered: (i) over a surface S atom (Figure S4a, Supporting Information), (ii) over a Cu atom (Figure S4b, Supporting Information), and (iii) over the C—N bond (Figure S4c, Supporting Information). The adsorption energies are almost the same for all three sites, with the latter being marginally more stable by less than 50 meV per C_60_ molecule. The optimized atomistic structure for the case of a C_60_ molecule adsorbed over a C—N bond is shown in Figure 4b. Upon adsorption of the C_60_, the charge rearrangement at the interface is minimal, and no formation of interfacial dipoles is observed. Consequently, the carrier injection and extraction are governed by the electron‐affinity rule. The band level alignment at the DFT‐Heyd‐Scuseria‐Erzerhof (HSE) level is shown in Figure 4c, and the details for the calculations are included in the Experimental Section. The level alignment is in agreement with the experimentally aligned energy levels shown in Figure 2c. We identify three prominent effects. First, the hole extraction from C_60_ is favored, as the CuSCN valence band top (VBT) is higher than the VBT of C_60_ by 0.5 V. Second, the conduction band bottom of CuSCN is 1.6 eV higher than C_60_, hence CuSCN will act as an efficient electron blocking layer. Finally, based on this level alignment, we can quantify the maximum ideal *V*_OC_ for a photovoltaic CuSCN‐C_60_ device to be at 1.7 V. We also performed another set of calculations where C_60_ was replaced with C_70_ and found that the band alignment remains unaffected.

Next, we calculate the electronic band structure and carrier effective masses for the bulk β‐CuSCN, shown in Figure 4d, and for solid C_60_ by employing the HSE hybrid functional. For CuSCN, the masses are very light along the molecular *c*‐axis (see Figure 4a) for both electrons (0.3*m*_e_) and holes (0.8*m*_e_). The latter value implies an even higher hole mobility value than those reported recently.^32^, ^33^, ^34^ To this end, experimental mobility determination should, ideally, be performed on the CuSCN single crystal where extrinsic carrier scattering effects are expected to be minimal. Along the plane perpendicular to the *c*‐axis, the masses are slightly heavier for electrons (2.1*m*_e_) and there are light and heavy holes with masses of 0.5*m*_e_ and 1.5*m*_e_, respectively. For solid C_60_, the effective masses of hole are isotropic (0.6*m*_e_–0.8*m*_e_) and in the same range with the electron masses along the direction that connects the first neighbors in the face‐centered cubic (FCC)‐arrangement (0.7*m*_e_). The similarity in the electron and hole masses is in agreement with the measured balanced electron and hole mobilities (Figure 1 and Table 1). Finally, the electron mass along the direction connecting the second neighbors is significantly heavier (4*m*_e_).

## 3 Conclusion

In conclusion, the efficient solar cells based on methanofullerenes, namely PC_60_BM and PC_70_BM, as the sole light absorbing material and CuSCN as the transparent hole‐extracting material, have been demonstrated. Although, the bilayer CuSCN/PC_70_BM devices were found to exhibit moderate performance with PCE of ≈1%, physical blending of the two components resulted in the solar cells with PCE of 5.4% and *V*_OC_ in excess of 0.9 V. Cells with PCE values of >6% were also fabricated, but proved to be difficult to reproduce reliably due to complex processing protocols employed. Analysis of the individual materials and device microstructures revealed that there are several factors that contribute to the high performance achieved, including: (i) the balanced and high mobility ambipolar charge transporting nature of the PC_60_BM and PC_70_BM derivatives, (ii) the superb hole‐transporting/electron‐blocking character of CuSCN, which helps to facilitate exciton dissociation and hole extraction at the critical CuSCN:fullerene interface, and (iii) the presence of a spontaneously formed mesostructured CuSCN‐nanowire:fullerene heterointerface—a truly hybrid organic/inorganic p–n interface unlike any other reported to date. First‐principle calculations confirm the beneficial nature of the CuSCN/fullerene interface for the charge separation. In fact, CuSCN not only acts as an efficient hole extracting layer but it also blocks the electron flow to the anode, resulting to large *V*_OC_ values. The significance of the present work is threefold: first, it conclusively shows that PC_60_BM and PC_70_BM are excellent ambipolar semiconductors, with highly balanced electron and hole mobilities; second, the solar cell data represent the highest reported PCE value for an organic solar cell based on a single light absorbing material; and third, it lays the foundation for an improved understanding of the charge photogeneration in the fullerene‐based solar cells where the role of the fullerene has been largely ignored. The latter may also underpins the widely reported beneficial role that fullerene interlayers play in the operating characteristics of the metal halide perovskite solar cells.

## Experimental Section

*Transistor Fabrication and Characterization*: 2 cm × 2 cm glass substrates were used for TG‐BC transistor fabrication. Before material depositions, the cleaning procedure for the substrates was carried out by sonication in detergent solution (DECON90), deionized water, acetone, and 2‐propanol (IPA) for 10 min each prior to use. 40 nm thick gold source and drain contacts were then thermally evaporated onto the cleaned substrates through shadow masks, defining the transistor channel length of 30 µm and the channel width of 1000 µm. The metal contact was modified with pentafluorothiophenol (PFBT) by immersing the samples into a 1:100 v/v% diluted solution of PFBT in IPA for 1 h at room temperature in air. The excess of PFBT was rinsed off with large quantity of IPA after removing the substrates from the diluted PFBT solution. The semiconductor layers using PC_70_BM (99%, Solenne) or PC_60_BM (99%, Solenne) based solution (both in chlorobenzene at concentration of 20 mg mL^−1^) were then spin‐cast at spin speed of 1200 rpm for 30 s onto the glass substrates, followed by a thermal‐annealing process at 100 °C for 10 min. The semiconductor deposition was taken place in a nitrogen filled glove box. The soluble fluoropolymer CYTOP (Asahi Glass) was used as the gate dielectric layer and spin‐cast on top of the PC_70_BM layer or the PC_60_BM layer at 2000 rpm for 60 s, followed by a drying process in a high vacuum chamber (≈10^−7^ bar) for 12 h. The device fabrication was completed with thermal evaporation of 40 nm Al gate electrode through shadow masks onto the gate dielectric. The electrical characterization of the transistors was carried out at room temperature in a nitrogen‐filled glove box using an Agilent B2902A parameter analyzer.

*Solar Cell Fabrication*: Indium tin oxide coated glass substrates (Kintec Company, 10 Ω per square) were cleaned by sequential ultrasonication in dilute Extran 300 detergent solution, deionized water, acetone, and isopropyl alcohol for 20 min each. These substrates were then cleaned by UV–ozone treatment for 30 min. Copper (I) thiocyanate (CuSCN) (25 mg mL^−1^) (Sigma‐Aldrich) was dissolved in diethyl sulfide (DES) (Sigma‐Aldrich) at 60 °C for 1 h. The CuSCN solution was then spin‐cast at 2500 rpm for 30 s, followed by annealing of the device at 105 °C for 10 min.

For the bilayer (CuSCN/PCBM) devices, PC_60_BM and PC_70_BM were dissolved in chlorobenzene (CB) at concentration of 30 and 20 mg mL^−1^, respectively. Both PC_60_BM and PC_70_BM layers were spun at 2000 rpm for 30 s (active‐layer thickness 30 nm), followed by thermal annealing at 105 °C. For the mixed layer, a blend layer of CuSCN:PC_70_BM was made by mixing 15 µL of CuSCN (40 mg mL^−1^ in DES) with 45 µL of PC_70_BM (40 mg mL^−1^ in CB) for 5 min before the spin‐coating process (for weight ratio of 1:3). The mixtures were then spin‐coated at 2000 rpm for 30 s, followed by thermal annealing at 105 °C. For CuSCN:PC_60_BM bulk heterojunction layer, a blend layer of CuSCN:PC_60_BM was made by mixing 10 µL of CuSCN (25 mg mL^−1^ in DES) with 40 µL of PC_60_BM (30 mg mL^−1^ in CB) 5 min before the spin‐coating process. Such mixture was then spin‐coated at 2000 rpm for 60 s, followed by thermal annealing at 105 °C. After the spin‐coating process, all substrates were then transferred to a vacuum chamber where 10 nm BCP (Sigma‐Aldrich) and 100 nm of aluminum were thermally evaporated at a base pressure of 5 × 10^−6^ mbar.

*Solar Cell Characterization: J–V* measurements of the solar cells were performed in a N_2_‐filled glove box using a Keithley 2400 source meter and an Oriel Sol3A Class AAA solar simulator calibrated to 1 sun, AM1.5G, with a KG‐5 silicon reference cell certified by Newport.

*External Quantum Efficiency (EQE) Measurement*: EQE was characterized using a specially designed EQE system (PV measurement Inc.). Measurements were performed at zero bias by illuminating the device with monochromatic light supplied from a Xenon arc lamp in combination with a dual‐grating monochromator. The number of photons incident on the sample was calculated for each wavelength by using a silicon photodiode calibrated by NIST.

*Subgap EQE Measurement*: The EQE spectra were collected at short‐circuit under focused monochromatic illumination from a Xenon arc lamp. The light beam was modulated by an optical chopper (275 Hz). The device output current was measured as a function of incident photon energy using a lock‐in amplifier (Stanford Instruments SR 830). The lamp intensity was calibrated with Ge and Si photodiodes.

*UV–VIS Absorption Measurements*: The optical absorption characteristics of the semiconductor blend layers deposited on quartz substrates were measured using a Varian Cary 5000 spectrophotometer. Active‐layer thicknesses were measured with a Tencor surface profilometer.

*High‐Resolution TEM Analysis*: For the imaging of the bilayer CuSCN/PC_70_BM and the mixed CuSCN:PC_70_BM cells' cross‐sections, the lamellae were prepared using a focused ion beam in a scanning electron microscope (Helios 400s, FEI) equipped with a nanomanipulator (Omniprobe, AutoProbe300). Here, sequential layers of carbon and platinum were deposited under electron and ion beams to protect the samples during the later stages of lamella preparation. Ga ion beam was used at 30 kV and 9 nA to mill the sample and then cut the lamella from the bulk. The lamella was mounted on the special TEM copper grid with the help of the nanomanipulator using the lift‐out method. The lamella was then thinned down with the ion beam (30 kV, 93 pA) and cleaned from the contamination at lower voltages (5 kV, 48 pA). The lamellae were then imaged with the TEM (Titan 80‐300, FEI) at the operating voltage of 300 kV. The EELS images were acquired in the same microscope with the Gatan Imaging Filter (GIF) Quantum 966.

*DFT Calculations*: All density functional theory calculations were performed using VASP.^61^ The HSE06 hybrid functional was employed with the short‐range separation parameter set at 0.2 Å^−1^.^62^ The projector augmented wave method^63^ was used with a 400 eV kinetic energy cutoff. For the structural optimization, the convergence threshold for the forces was set to 0.01 eV Å^−2^. The Brillouin zone was sampled by employing 2 × 2 × 1 Г‐centered Monkhorst‐pack grids. To model the (11$\bar{2}$0) CuSCN surface, a slab consisting of ten molecular layers and at least 15 Å of vacuum separating the periodic images was used. The atomic coordinates of the slab using the experimental lattice parameters (*a* = 3.85 Å and *c* = 10.938 Å) of the wurtzite β‐phase of CuSCN was optimized. To accommodate the C_60_ molecules, a 2 × 1 tetragonal supercell was used. The center‐to‐center distance of the fullerenes on the super‐cell is 13.34 and 10.94 Å, which are slightly larger than the corresponding distance in FCC‐C_60_ (≈10 Å).^64^ The band alignment was calculated at the DFT‐PBE level and corrected by the HSE06 values from the calculations on the bulk β‐CuSCN and C_60_ systems.

## Conflict of Interest

The authors declare no conflict of interest.

## Supporting information

Supplementary
