# Supplementary material for: High‐Efficiency Fullerene Solar Cells Enabled by a Spontaneously Formed Mesostructured CuSCN‐Nanowire Heterointerface
Source: Adv Sci (Weinh). 2018 Feb 2;5(4):1700980. doi: 10.1002/advs.201700980 (PMC5908360; doi:10.1002/advs.201700980)
Supplement: Supplementary file 1 — Supplementary [file ADVS-5-1700980-s001.pdf]

Copyright WILEY-VCH Verlag GmbH & Co. KGaA, 69469 Weinheim, Germany, 2018.

ADVANCED SCIENCE
Open Access

Supporting Information

for *Adv. Sci.,* DOI: 10.1002/advs.201700980

High-Efficiency Fullerene Solar Cells Enabled by a Spontaneously Formed Mesostructured CuSCN-Nanowire Heterointerface

*Wai-Yu Sit, Flurin D. Eisner, Yen-Hung Lin, Yuliar Firdaus, Akmaral Seitkhan, Ahmed H. Balawi, Frédéric Laquai, ClaireH. Burgess, Martyn A. McLachlan, George Volonakis, Feliciano Giustino, and Thomas D. Anthopoulos**

Copyright WILEY-VCH Verlag GmbH & Co. KGaA, 69469 Weinheim, Germany, 2016.

Supporting Information

High efficiency fullerene solar cells enabled by a spontaneously formed mesostructured CuSCN-nanowire heterointerface

Wai-Yu Sit, Flurin Eisner, Yen-Hung Lin, Yuliar Firdaus, Akmaral Seitkhan, Ahmed H. Balawi, Frédéric Laquai, Claire Burgess, Martyn A. McLachlan, George Volonakis, Feliciano Giustino, and Thomas D. Anthopoulos

Table S1. Summary of the mean extracted parameters from bilayer and mixed layer cells under AM1.5 illumination. The values for the best cell are given in brackets.

	V_{OC} [V]	J_{SC} [mAcm^{-2}]	*FF* [%]	*PCE* [%]
$PC_{70}BM$/CuSCN (bilayer)	0.98 ± 0.030 (0.95)	1.8 ± 0.24 (2.2)	48 ± 3.2 (49)	0.84 ± 0.12 (1.0)
$PC_{70}BM$:CuSCN (mixed layer)	0.91 ± 0.005 (0.91)	7.9 ± 0.11 (8.3)	71 ± 1.9 (72)	5.10 ± 0.17 (5.4)

Table S2. Solar cell performance dependence on the $PC_{70}BM$ thickness for bilayer cells (top table) and the performance dependence on the CuSCN:$PC_{70}BM$ ratio of mixed layer cells for two different total solid concentrations (bottom table).

Active layer		V_{OC} [V]	J_{SC} [mA cm^{-2}]	*FF* [%]	*PCE* [%]
CuSCN/$PC_{70}BM$ (bilayer)					
30 nm		0.98 ± 0.03	1.8 ± 0.24	48 ± 3.2	0.84 ± 0.12
50 nm		0.96 ± 0.01	1.7 ± 0.01	43 ± 1.0	0.70 ± 0.03
85 nm		0.94 ± 0.02	0.5 ± 0.09	39 ± 2.3	0.18 ± 0.04
105 nm		0.93 ± 0.02	0.36 ± 0.05	40 ± 5.3	0.13 ± 0.04
CuSCN:$PC_{70}BM$ (mixed layer)					
Total solid: 25 mg/ml	1:5	0.93 ± 0.004	5.9 ± 0.15	71 ± 1.6	3.9 ± 0.15
	1:3	0.92 ± 0.007	7.1 ± 0.06	73 ± 0.9	4.7 ± 0.07
	1:2	0.92 ± 0.004	7.6 ± 0.12	72 ± 1.7	5.0 ± 0.08
Total solid: 40 mg/ml	1:5	0.92 ± 0.008	7.0 ± 0.11	71 ± 1.2	4.6 ± 0.12
	1:3	0.91 ± 0.005	7.9 ± 0.11	71 ± 1.9	5.1 ± 0.17
	1:2	0.92 ± 0.008	7.9 ± 0.08	67 ± 3.1	4.8 ± 0.16

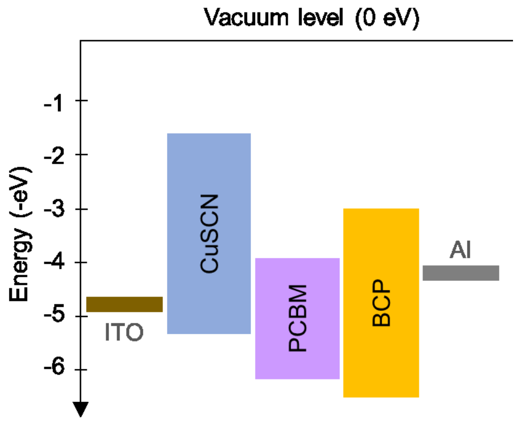

Figure S1. Energy levels of the various materials used to fabricate solar cells.

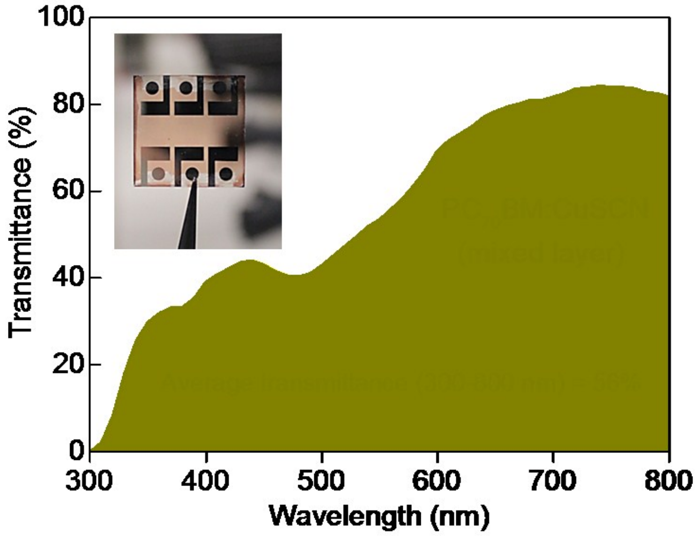

Figure S2. Transmission spectra of an optimized CuSCN:$PC_{70}BM$ mixed layer device measured without the top Al electrode. The active layer of the device exhibits an average transmittance between 300-800 nm of 56%. Inset: photograph of the actual device.

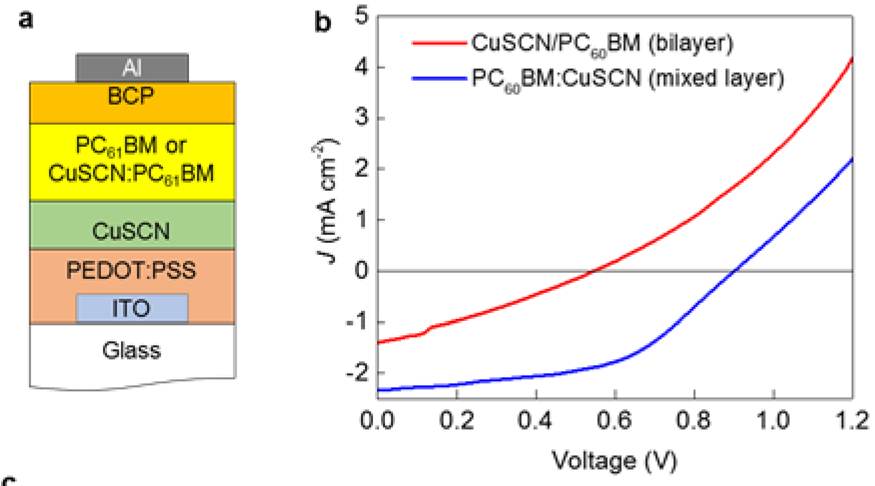

c

Active Layer	J_{sc} [mAcm^{-2}]	V_{oc} [V]	*FF* [%]	*PCE* [%]
CuSCN:PC_{60}BM (mixed layer)	2.3	0.90	51	1.1
CuSCN/PC_{60}BM (bilayer)	1.4	0.55	29	0.22

Figure S3. (a) Device architecture of the CuSCN:PC_{60}BM solar cells. (b) *J-V* characteristics measured for a CuSCN/PC_{60}BM bilayer and a CuSCN:PC_{60}BM mixed layer solar cells. (c) Summary of key device parameters extracted from the *J-V* characteristics.

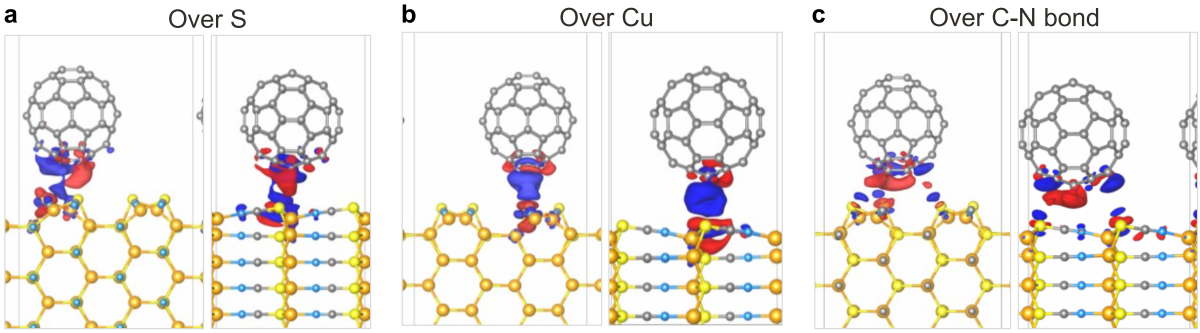

Figure S4. Atomistic model and charge density re-arrangement of the adsorption of a C_{60} molecule onto CuSCN viewed from the top (left) and side on (right) over three different possible adsorption sites: a) over a surface S atom, (b) over a Cu atom and (c) over the C-N bond. The calculated adsorption energies are almost the same for all three sites. Blue (red) shows the positive (negative) charge redistribution upon absorption of the C_{60}.

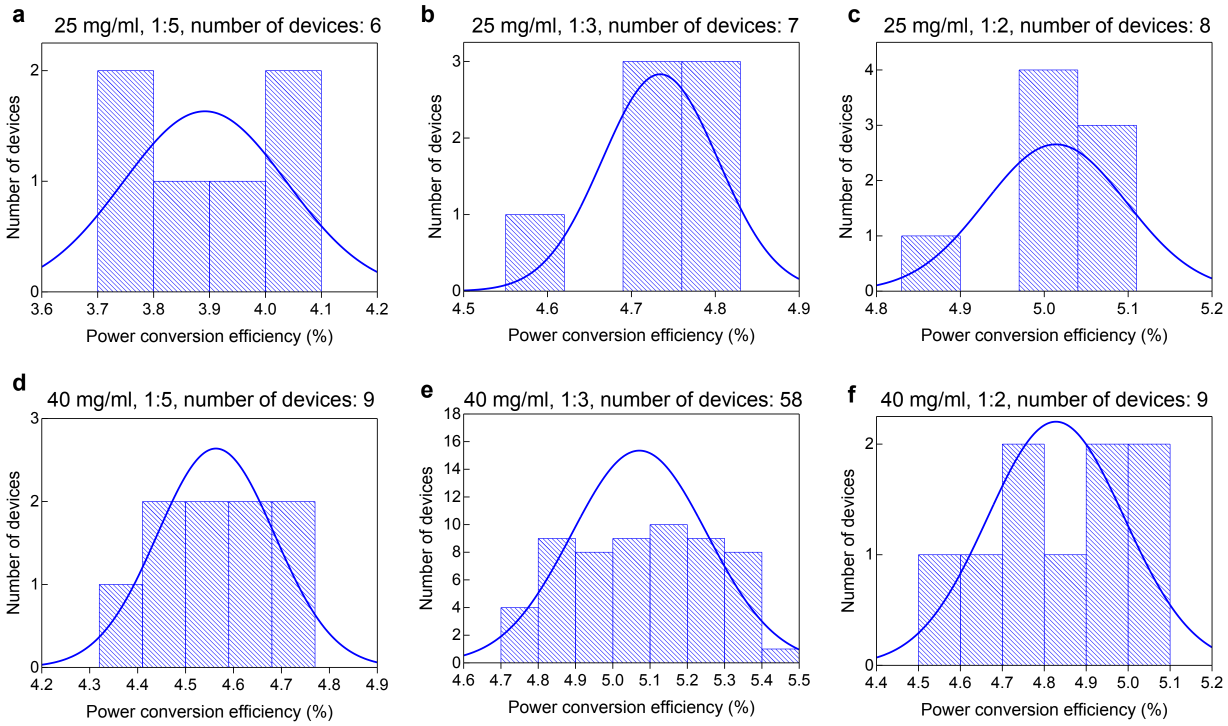

Figure S5. (a-f) Plots of the statistical device power conversion efficiency (PCE) versus number of devices for the data of the mixed-layer cells of different CuSCN:PCBM compositions corresponding to the values shown in Table S2 (bottom). The total solid concentration, followed by the ratio of CuSCN to $PC_{70}BM$ content and the total number of devices tested are indicated above each plot.

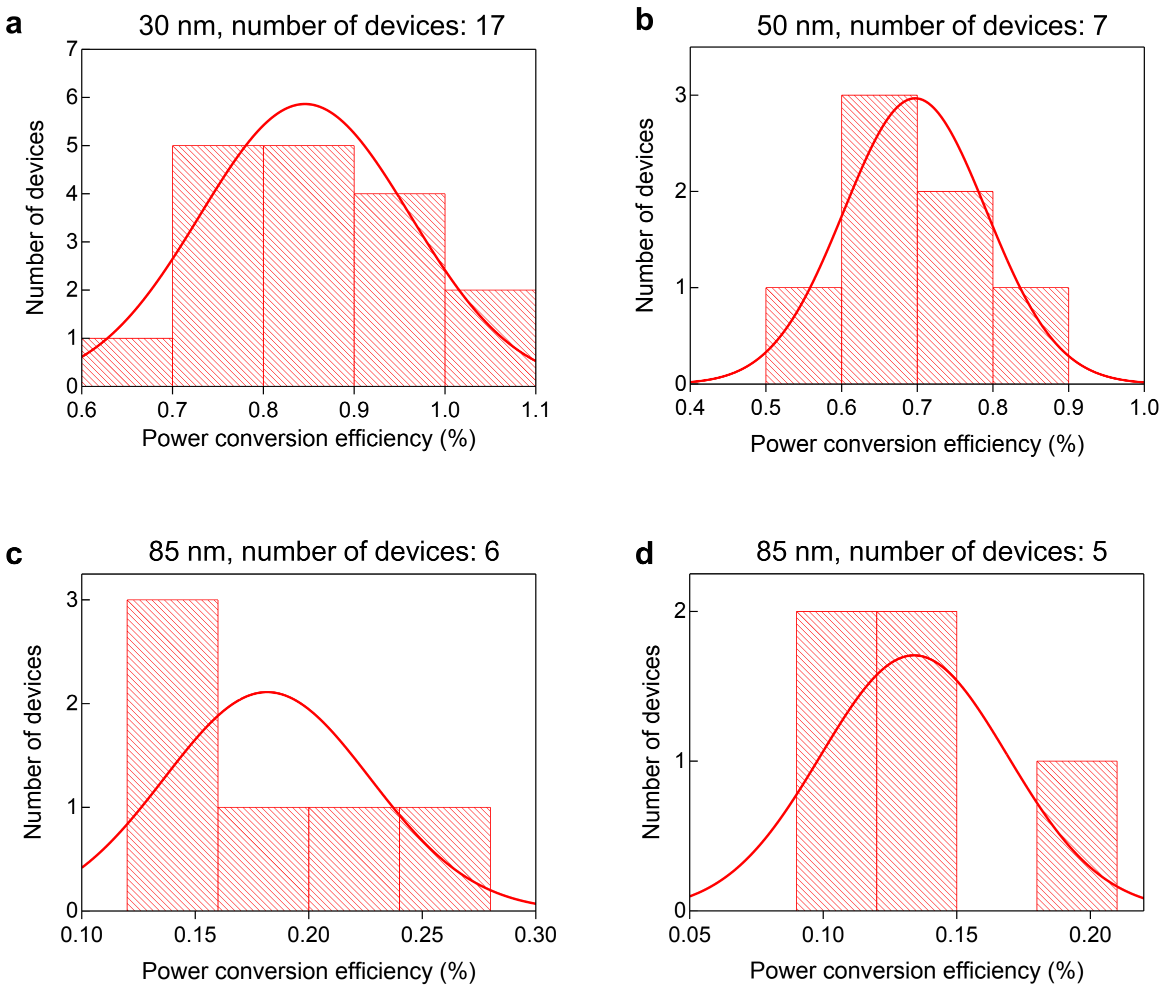

Figure S6. (a-d) Plots of the statistical device power conversion efficiency (PCE) versus number of devices for the data of the bilayer cells of different thicknesses corresponding to the values shown in Table S2 (top). The $PC_{70}BM$ thickness followed by the total number of devices tested is indicated above each plot.
